# Random species loss underestimates dilution effects of host diversity on foliar fungal diseases under fertilization

**DOI:** 10.1002/ece3.3749

**Published:** 2018-01-08

**Authors:** Xiang Liu, Fei Chen, Shengman Lyu, Dexin Sun, Shurong Zhou

**Affiliations:** ^1^ State Key Laboratory of Earth Surface Processes and Resource Ecology Beijing Normal University Beijing China; ^2^ Ministry of Education Key Laboratory for Biodiversity Science and Ecological Engineering School of Life Sciences Fudan University Shanghai China

**Keywords:** alpine meadows, biodiversity–disease relationships, experimental warming, nitrogen addition, nonrandom diversity loss, Qinghai‐Tibetan Plateau

## Abstract

With increasing attention being paid to the consequences of global biodiversity losses, several recent studies have demonstrated that realistic species losses can have larger impacts than random species losses on community productivity and resilience. However, little is known about the effects of the order in which species are lost on biodiversity–disease relationships. Using a multiyear nitrogen addition and artificial warming experiment in natural assemblages of alpine meadow vegetation on the Qinghai‐Tibetan Plateau, we inferred the sequence of plant species losses under fertilization/warming. Then the sequence of species losses under fertilization/warming was used to simulate the species loss orders (both realistic and random) in an adjacently novel removal experiment manipulating plot‐level plant diversity. We explicitly compared the effect sizes of random versus realistic species losses simulated from fertilization/warming on plant foliar fungal diseases. We found that realistic species losses simulated from fertilization had greater effects than random losses on fungal diseases, and that species identity drove the diversity–disease relationship. Moreover, the plant species most prone to foliar fungal diseases were also the least vulnerable to extinction under fertilization, demonstrating the importance of protecting low competence species (the ability to maintain and transmit fungal infections was low) to impede the spread of infectious disease. In contrast, there was no difference between random and realistic species loss scenarios simulated from experimental warming (or the combination of warming and fertilization) on the diversity–disease relationship, indicating that the functional consequences of species losses may vary under different drivers.

## 1 INTRODUCTION

Accelerating biodiversity losses as a result of increasing anthropogenic pressures may result in the alteration of a range of ecosystem functions and services (Chapin et al., 2000; Naeem, Duffy, & Zavaleta, 2012), as many previous studies have revealed causal relationships between species richness and productivity (Hector, Bazeley‐White, Loreau, Otway, & Schmid, 2002), stability (Tilman, Reich, & Knops, 2006), invasibility (Fargione & Tilman, 2005), and the severity of infectious diseases (Mitchell, Reich, Tilman, & Groth, 2003). Using randomly constructed species assemblages, most manipulative biodiversity–ecosystem function (BEF) experiments have evaluated the effects of species number *per se* on ecosystem functions. Such experiments have greatly benefited ecological theory and expanded our knowledge base (Fargione & Tilman, 2005; Tilman, Isbell, & Cowles, 2014; Tilman et al., 2001, 2006).

However, the underlying assumption of BEF experiments using randomly assembled communities is that all species have equal probability of local extinction (Selmants, Zavaleta, Pasari, & Hernandez, 2012), whereas species losses are often nonrandom with respect to particular traits or phylogeny in real ecosystems (Zavaleta et al., 2009). Recently, several studies have demonstrated that there are larger functional consequences of realistic versus random species losses on biomass production (Wolf & Zavaleta, 2015), invasion resistance (Selmants et al., 2012; Zavaleta & Hulvey, 2004, 2007), and nitrogen‐use (Bracken, Riberg, Gonzalezdorantes, & Williams, 2008; Selmants, Zavaleta, & Wolf, 2014). These studies emphasize the influence of species composition, as well as the specific order of species losses, on ecosystem functions, a factor that is often overlooked in BEF studies. The observed discrepancy in diversity effect sizes between realistic and random species loss scenarios possibly arises from the correlation between a species’ vulnerability (to loss) and its importance to certain ecosystem functions (Zavaleta et al., 2009).

It has been well documented that biodiversity can affect the emergence and severity of infectious diseases (Cardinale et al., 2012; Johnson, Preston, Hoverman, & Richgels, 2013; Keesing et al., 2010). Higher biodiversity may decrease the emergence and transmission of infectious diseases, thus providing a “dilution effect” as an important ecosystem service, or it may instead amplify the emergence and spread of diseases (Civitello et al., 2015; Johnson, Ostfeld, & Keesing, 2015; Keesing, Holt, & Ostfeld, 2006; Ostfeld & Keesing, 2012). Field studies in both natural and artificial ecosystems (mainly random species loss experiments; e.g., Hantsch, Braun, Scherer‐Lorenzen, & Bruelheide, 2013; Hantsch et al., 2014; Knops et al., 1999; Liu, Lyu, Zhou, & Bradshaw, 2016; Mitchell, Tilman, & Groth, 2002; Rottstock, Joshi, Kummer, & Fischer, 2014), and also meta‐analysis (Civitello et al., 2015), have overwhelmingly documented dilution rather than amplification effects; however, the underlying mechanisms remain unclear. Furthermore, although the randomized species losses in these experiments isolated the effect of species number *per se* on disease prevalence, avoiding confounding effects of species identity (Huston, 1997), randomized species lose almost does not occur in natural conditions. There is accumulating evidence of a positive relationship between a host's competence (its ability to maintain and transmit infections) for pathogens and its ubiquity (i.e., its presence across sites of varying species diversity; Johnson et al., 2013; Lacroix et al., 2014), calling the experimental premise into question. In the case of a competence–ubiquity relationship, more common species would have larger effects not only on community disease severity (pathogen load), but also on the biodiversity–disease relationship. If the poorly competent host species was the most at risk of loss in community disassembly, the host communities that were initially dominated by less competent hosts will transform to highly competent ones, which would cause a steeper biodiversity–disease relationship than expected under random species losses. However, whether or not realistic versus random species losses produce different effects on pathogen load (hereafter referred to simply as the “effect size”) has not been explicitly investigated.

In particular, the functional consequences of random versus realistic species losses simulated from identified global change drivers (e.g., drought, warming, nitrogen deposition, invasion, land‐use intensification, etc.) remain largely unstudied. Looking specifically at infectious diseases, a negative relationship between a host species’ vulnerability to these drivers and its competence may result from evolutionary trade‐offs among host competitive ability, growth, and disease resistance (Huang et al., 2013), as both competition and defense traits can be costly (especially for defense against specialist enemies; Leibold, 1989; Viola et al., 2010). When such trade‐offs occur, studies using random species losses may fail to accurately predict disease severity under diversity decline or to provide correct implications for conservation. For example, numerous nitrogen addition experiments have demonstrated that fertilization can lead to biodiversity decline via exclusion of competitively inferior species (mainly via light competition; Suding et al., 2005; Hautier, Niklaus, & Hector, 2009; Yang, Hautier, Borer, Zhang, & Du, 2015), while experimental warming (simulated by open‐top chambers) was shown to lead to rapid species loss in alpine meadows owing to heat stress, warming‐induced drought, and litter accumulation (Klein, Harte, & Zhao, 2004; Liu et al., 2016). Hence, species loss orders simulated from nitrogen addition and warming may have different effects on biodiversity–disease relationships because of the different mechanisms causing species losses under these two drivers.

Here, we used data collected in a multiyear fertilization and artificial warming experiment to infer the sequence of real species losses under nitrogen addition and experimental warming. Then the sequence of species losses under fertilization/warming was used to simulate the species loss orders (both realistic and random) in a field‐based removal experiment in alpine meadows on the Qinghai‐Tibetan Plateau, a system that is largely underinvestigated and vulnerable to disturbance (Li et al., 2014; Yang et al., 2015). We focused on foliar fungal pathogens, which are largely specific to a certain host species and which represent the most important diseases in alpine meadows (Zhang, 2009). Hence, these specialist pathogens represent an ideal plant‐fungal system to test for different effects of realistic (simulated from fertilization or warming) versus random species losses on biodiversity–disease relationships. Specifically, we tested the following predictions: (1) increasing host species richness alters the severity of foliar fungal diseases, either negatively (dilution effect) or positively (amplification effect), regardless of the order of species losses; (2) host species with good defense capabilities (low fungal infections in natural plots) would be the most at risk of loss under fertilization, instead of warming; which would cause (3) a steeper biodiversity–disease relationship under realistic species loss orders simulated from fertilization than that under random species losses.

## 2 MATERIALS AND METHODS

### 2.1 Study site

This study was conducted at the Alpine Meadow and Wetland Ecosystems Research Station of Lanzhou University, which is located in Maqu, Gansu Province, on the eastern part of the Qinghai‐Tibetan Plateau of China (101°53′E, 35°58′N, 3,500 m a.s.l.). Maqu has a mean annual precipitation of 620 mm, with most of the precipitation occurring during the growing season (June–August). Mean annual temperature is 1.2°C, with the monthly average ranging from −10.7°C (January) to 11.7°C (July). The nitrogen‐limited soils have a mean thickness of 80 cm and are classified as subalpine meadow soils according to the Chinese soil classification system. The grassland vegetation comprises typical alpine meadow species, dominated by perennial herbs in the Poaceae, Asteraceae, Ranunculaceae, and Fabaceae families, such as *Anemone rivularis*,* Elymus nutans*,* Festuca sinensis*, and *Ligularia virgaurea* (see Table S1 for a species list). The dominant animal species include yaks, marmots (*Marmota himalayana*), and a number of ant species (Liu et al., 2015).

### 2.2 Experimental design

A roughly 100 × 200 m study site was enclosed with a fence in 2009, with grazing (mainly yaks) only permitted in winter (consisting with traditional grazing style). We used data collected in a fertilization and artificial warming experiment (5 years of experimental nitrogen addition and 4 years of warming) to infer the order of real species losses under nitrogen addition and experimental warming. This experiment was established in June 2011. It consisted of 48 regularly arranged 5 × 5 m plots separated by 1 m (buffer zone) from adjacent edges and also each other. All 48 plots were randomly assigned to one of four concentrations of nitrogen addition: 0 (control), 5, 10, or 15 g/m^2^. So there were 12 replicates of each nitrogen treatment, and a half of the 12 replicates (6 plots) of each nitrogen treatment were warmed using transparent, reinforced‐plastic, and open‐top chambers (OTCs) with a 1.5 m^2^ basal area placed at the centre of the plot (there was a total of 6 × 4 = 24 warming plots). Nitrogen was supplied as ammonium nitrate (NH_4_NO_3_), and fertilizer was broadcast evenly in each plot once per year in mid‐June (i.e., early in the growing season) from 2011; fertilizer was only broadcast when the weather was cloudy or rainy. Meanwhile, from 2011 to 2014, we placed OTCs in warming plots in early May and removed them in early October annually (4 years in total). On average, the OTCs increased the air temperature of the experimental warming plots by 0.77**°**C at night and by 1.8**°**C during the day. As for soil temperature, at a depth of 10 cm, it increased by ~0.73**°**C during the day and remained nearly unchanged at night (Liu et al., 2016).

According to our previous study, host plant species richness decreased with fertilization linearly. There were on average 30 host plant species in the natural plots, while only about 20 species in the 15 g/m^2^ fertilization plots, and about 15 species in the warming plots (Liu, Lyu, Sun, Bradshaw, & Zhou, 2017; Liu et al., 2016). Host plant species with lower height, higher nitrogen content, and also lower fungal infections in natural condition (e.g., *Leguminosae*) were the most at risk of loss under fertilization, which indicates a trade‐off between host competitive ability and fungal infections (defense system; Liu et al., 2017).

We then established the species removal experiment in the southeast‐facing meadow with little slope in June 2014 (Figure S1), which was adjacent to the aforementioned fertilization/warming experiment (only 5 m apart). The removal experiment consisted of 120 regularly arranged, 1.5 × 1.5 m square plots separated by 3 m (buffer zone) from adjacent edges and also each other. A full description of this removal experiment is provided in Liu et al. (2016, 2017), but we provide a brief introduction here. We selected 12 herbaceous species with similar abundances at the study site for our experiments to avoid any density effects on community pathogen load. In total, these 12 species included four grasses, two legumes and six nonleguminous herbaceous forbs (Table 1). The 120 plots were randomly assigned to a species richness treatment of either one (36 plots, with three replicates for each species), two (24 replicates), four (24 replicates), eight (24 replicates), or a control (12 replicates). For richness treatment levels of two, four and eight species, we randomly selected species from the 12 species, and then removed all other species by clipping all aboveground biomass (and damaging the root as much as possible by digging deeply) appearing in the plot artificially twice a year: firstly in late May/early June (the beginning of growing season) and second in early July 2014 and 2015, respectively. For the 36 monocultures, we maintained the focal species and removed (by clipping) all the other species in the same manner.

**Table 1:** Twelve host species used in the removal experiment. Shown are the sequence in which species were lost under fertilization (frequency data) and warming, the disease susceptibility index, and the functional groups

Species	Loss sequence under fertilization	Loss sequence under warming	Disease Susceptibility index	Functional group
*Ligularia virgaurea*	IV‐12	IV‐10	7.546	Perennial forb
*Anemone rivularis*	IV‐11	III‐8	7.283	Perennial forb
*Elymus nutans*	IV‐10	I‐3	3.168	Perennial grass
*Kobresia humilis*	III‐9	I‐2	1.492	Perennial grass
*Saussurea stella*	III‐8	III‐9	1.694	Annual forb
*Thermopsis lanceolala*	III‐7	III‐7	2.708	Perennial N‐fixer
*Anemone trullifolia*	II‐6	IV‐11	3.253	Perennial forb
*Potentilla potaninii*	II‐5	IV‐12	2.508	Perennial forb
*Saussurea nigrescens*	II‐4	II‐5	4.837	Perennial forb
*Koeleria litvinowii*	I‐3	I‐1	2.444	Perennial grass
*Festuca sinensis*	I‐2	II‐4	1.250	Perennial grass
*Astragalus polycladus*	I‐1	II‐6	0.750	Perennial N‐fixer

“I” means the most, while “IV” means the least, vulnerable to loss under nitrogen addition or warming.

### 2.3 Sampling

For each plot in the removal experiment, we randomly arranged three 0.2 × 0.5 m subplots parallel to one edge of the plot and at least 0.1 m away from the edges in August 2015. We then harvested all the stems in each subplot at ground level, sorted to species, recorded each species abundance; and then dried and weighed them to an accuracy of 0.1 mg as biomass. For each nitrogen addition plot, we randomly arranged a 0.5 × 0.5 m subplot parallel to one edge of the plot and at least 1 m away from the edges, and then assessed the abundance and biomass of each species in the subplot annually (in August) from 2011. We used the same approach for the artificial warming plots, using a single 0.5 × 0.5 m subplot to survey the sequence of species losses from 2011 to 2014 under experimental warming.

A full description of the method used to estimate disease severity in the experimental plant communities is provided in Liu et al. (2016), but we provide a brief synopsis here. We recorded foliar fungal disease severity on leaf replicates (see below; Mitchell et al., 2002) in August 2015 (peak growing season) for the removal experiment (not fertilization/warming experiment), as some fungal diseases (e.g., rust) can be found only in August. For each host plant species in each plot, we recorded disease severity for 25 leaves, with five leaves from each of five randomly selected individuals. We also collected three samples of infected plant tissue per plant species in July 2015 and confirmed the taxa of the pathogen (whether a fungal, bacterial, or viral disease) in the laboratory using an OLYMPUS light microscope (see Table S2 for the preliminary results). In order to get enough samples to calculate a disease susceptibility index (see below), we also recorded disease severity using the same approach for an additional 20 nonexperimental plots (0.5 × 0.5 m; 10 m spacing among plots) at the same study site (which was adjacent to both fertilization/warming experiment and removal experiment) with same management (also fenced since 2009).

### 2.4 Realistic and randomized loss orders

**Figure 1:**
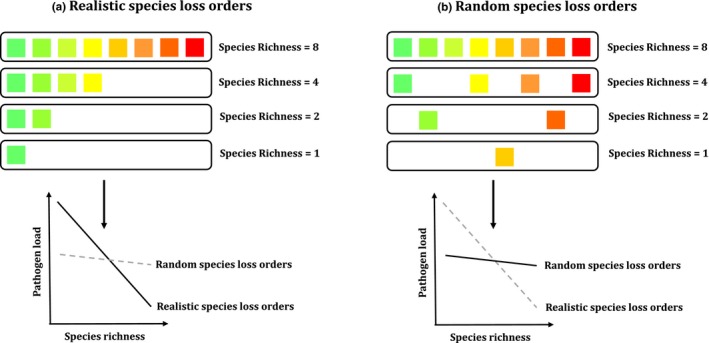
Hypothetical species loss orders under the scenarios of realistic and random extinction, and the predicted patterns of biodiversity–disease relationships. (a) Realistic species loss orders, resulting in a steeper biodiversity–disease relationship than random species losses. (b) Random species loss orders. The black boxes represent plots in the removal experiment, with species richness equal to 1, 2, 4, and 8, respectively. The squares represent plant species, and red indicates plant species with a high risk of loss under fertilization/warming than the green ones

Based on the sequence of plant species losses inferred from fertilization/warming, we simulated both the realistic and random species loss orders (i.e., we “selected” realistic and random species loss orders from a 120 plot pools) based on the removal experiment. We determined realistic sequence of species losses according to the following rules: (1) nestedness: plots at each species richness level must contain a subset of the species in plots of the next highest species richness level; and (2) species loss orders (simulated from fertilization and warming respectively): as determined directly from the multiyear nitrogen addition and artificial warming experiments. Each order of species losses we selected includes four plots: from host plant species richness treatment equal to 1, 2, 4, and 8, respectively. A plot can be used repeatedly to create species loss orders, so a certain plot can appear in different order of species losses, both realistic and random ones. For the nitrogen addition treatment, to avoid any fluctuations in the plant community structure among years due to exogenous factors such as temperature and precipitation, we sequenced the 12 species used in the removal experiment based on their frequency (in the plots) across different nitrogen addition levels in 2015, and then divided them equally into four groups of vulnerability to loss [see Table 1: e.g., group “I” was the most vulnerable to loss under nitrogen addition (i.e., red ones in Figure 1), while group “IV” was the least (i.e., green ones in Figure 1)] and we regressed the species’ vulnerability of each host species on the sequence in which it was lost (i.e., whether first, second, third, etc.). In the warming experiment, there was neither enough temperature variability across plots nor enough replication to calculate the frequency of the 12 species with progressive warming; hence, we recorded the sequence of species losses chronologically (from 2011 to 2014) for each artificial warming plot, and warming and fertilization interaction plot. Averaging over plots of the same type, we then determined the average sequence of species loss and divided species equally into four group of susceptibility to loss as before.

To construct realistic plant communities for the removal experiment, for each species richness level, we always selected a group with more vulnerable (to loss) species (i.e., red ones in Figure 1) for the next highest species richness level (Figure 1a). Exclusively, for plots with a species richness of eight, plant communities with one or two species belonging to group “III” were selected to provide enough realistic species losses. This made for “realistic” species loss orders among plots because such scenarios were seen to actually happen in our nitrogen addition and warming experiments (see Table S3 for all realistic species loss orders simulated from fertilization/warming treatments). Finally, we randomly selected plots from each species richness level to generate a set of randomized species loss orders (Figure 1b).

For each generated species loss order, we calculated the matrix temperature (*T*; Rodríguez‐Gironés & Santamaria, 2006), where low values of *T* equate to a high degree of nestedness (Atmar & Patterson, 1993). We repeated the calculation of *T* 500 times for each constructed loss order and took the average value as the mean *T* (Wolf & Zavaleta, 2015). In 2015, *T* = 25.24 (*p *<* *.001) for the nitrogen addition plots, while *T* ranged from 15.10 to 24.66 for the artificial warming plots, indicating a relatively strong nested structure (i.e., lower diversity treatments were subsets of higher diversity treatments; Wolf & Zavaleta, 2015).

### 2.5 Measures of disease severity

To make the biodiversity–disease relationships under realistic and random species loss orders comparable, measures of disease severity were based on samplings in the removal experiment. We defined a “severity index” (*V*_*i*_) as the average proportion of leaf area for a given plant species infected by disease *i*, and calculated the community abundance weighted mean of *V* for each species, which is equivalent to the “pathogen load” (*l*; Liu et al., 2016; Mitchell et al., 2002).

In order to characterize variation in susceptibility to foliar fungal diseases among different host species, we also defined a “disease susceptibility index” (*P*_*i*_) as the average severity index (*V*_*i*_) in the 32 nonmanipulated plots (12 controls plus 20 additional nonexperimental plots) for a specific plant species infected by disease *i*.

### 2.6 Analysis

The slope of the diversity–pathogen load relationship was determined for each simulated species loss order (both realistic and randomized), with pathogen load as the response variable and species richness as the independent variable in the linear model. We refer to this slope (the regression coefficient) as the diversity effect size on pathogen load (hereafter “effect size”) for each constructed species loss order. A negative effect size means a dilution effect of biodiversity on pathogen load, while a positive relationship means an amplification effect. We used *t* tests to assess the difference in effect size between realistic and random species losses. We fit a linear relationship between species richness (log‐transformed) and pathogen load for both realistic and random scenarios to illustrate how effect size differed between the scenarios.

We fit linear models to test the relationships between disease susceptibility index and the loss sequence for the 12 host species under fertilization and warming, respectively. Furthermore, when we fit linear models to test relationships in different scenarios, we calculated the information‐theoretic Akaike's information criterion corrected for small sample sizes (AIC_c_), wAIC_c_ *,* and the information‐theoretic evidence ratio (ER, wAIC_c_[slope model]:wAIC_c_[intercept‐only model]) ER as an index of relative support for the linear slope model (Burnham, Anderson, & Huyvaert, 2011). When ER > 1.5, the model has high support. We also calculated percent deviance explained (De) in the response variable as an index of each model's goodness of fit. We did all analyses in R 2.15.1 (R Development Core Team 2014).

## 3 RESULTS

**Figure 2:**
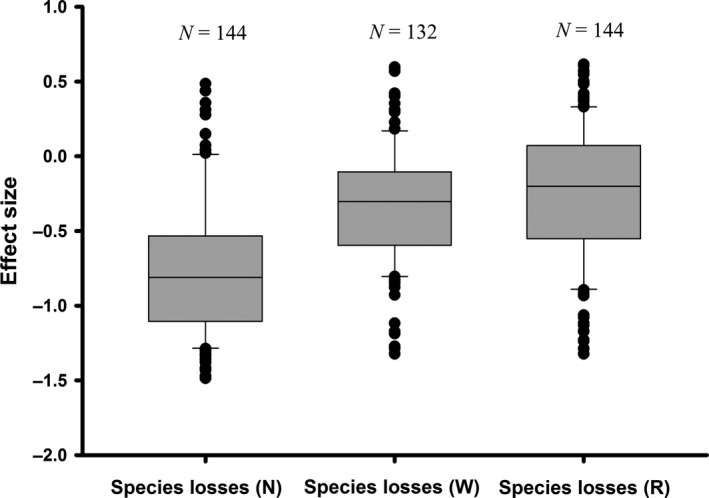
Differences in effect size (i.e., the slope of the regression between species richness and pathogen load) between random (R) and realistic [simulated from fertilization (N) and warming (W), respectively] species loss orders. (a) Mean effect size of realistic species losses simulated from fertilization (−0.747 ± 0.039) was greater than the random mean effect size (−0.257 ± 0.038; *t *=* *9.114, *p *<* *.001). (b) Mean effect size of realistic species losses simulated from fertilization (−0.747 ± 0.039) was greater than that simulated from warming (−0.322 ± 0.034; *t *=* *8.255, *p *<* *.001). (c) No difference in mean effect size between random species losses (−0.257 ± 0.038) and realistic species losses simulated from warming (−0.322 ± 0.034; *t *=* *1.280, *p *=* *.202)

**Figure 3:**
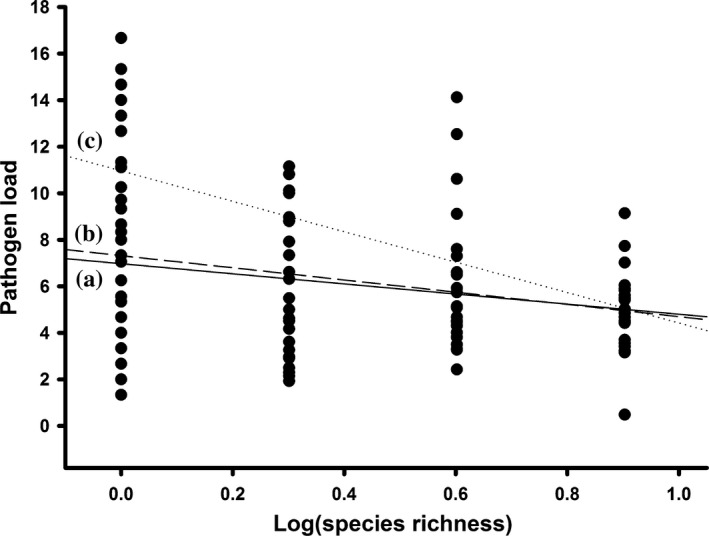
Pathogen load versus species richness for random and realistic species loss scenarios. (a) Random species losses: *y* = 6.977 − 2.175*x*; information‐theoretic evidence ratio (ER) = 5.34 × 10^6^; percent deviance explained (De) = 5.57. Pathogen load decreased significantly with species richness overall: ER = 12.95; De = 6.42. (b) Realistic species losses simulated from warming: *y* = 7.408 − 2.764*x*; ER = 4.45 × 10^18^; De = 14.15; (c) Realistic species losses simulated from fertilization: *y* = 10.964 − 6.543*x*; ER = 1.536 × 10^61^; De = 38.90. Each point represents a plot in the removal experiment (*n* = 108 plots, 12 control plots not included)

Based on simulating approach, realistic species loss order simulated from fertilization showed greater effects of biodiversity on foliar fungal disease severity (i.e., greater dilution effects) than random losses (*t *=* *9.11; *p *<* *.001; Figure 2). The mean effect size of realistic species losses simulated from fertilization was −0.747 ± 0.039, while this value increased to −0.257 ± 0.038 for random species losses. In contrast, the difference in mean effect size between realistic species losses simulated from experimental warming (−0.322 ± 0.034) and random species losses (*t *=* *1.28, *p *=* *.202) was not significant. Although pathogen load always decreased significantly with species richness (i.e., a dilution effect), random species losses largely underestimated the dilution effect of host diversity on fungal diseases under fertilization (Figure 3).

Under the combined effects of warming and fertilization where species loss order was determined chronologically from 2011 to 2014, at rates of 5, 10, and 15 g/m^2^ of nitrogen addition, experimental warming decreased the magnitude of effect sizes in realistic species loss scenarios consistently (Table 2). Furthermore, when determining species loss order chronologically from 2011 to 2014 in fertilization plots, effect size differed marginally between random species loss scenarios (−0.257 ± 0.038) and realistic (−0.357 ± 0.041 of 5 g/m^2^ and −0.346 ± 0.038 of 10 g/m^2^, respectively) species loss scenarios simulated from fertilization alone at 5 and 10 g/m^2^ of nitrogen addition (*p *=* *.074 and *p *=* *.098, respectively). However, at these same levels of nitrogen addition, effect sizes did not differ between realistic and random species loss scenarios under warming and fertilization combined (*p *=* *.244 and *p *=* *.665, respectively). Nonetheless, simulated from both fertilization alone and warming and fertilization combined, realistic species losses had greater dilution effects (−0.576 ± 0.045 and −0.570 ± 0.047, respectively) at 15 g/m^2^ of nitrogen addition than random species losses (−0.257 ± 0.038).

**Table 2:** Results of *t*‐tests comparing effect sizes between random species losses and realistic species losses simulated from various treatments: warming treatment (W), nitrogen fertilization treatment (N) and combination of nitrogen fertilization and warming treatment (W × N). The sequence of species losses was sequenced chronologically from 2011 to 2014

Treatment	*N*	Mean ± *SE*	*t*	*p*
N (5 g)	129	−0.357 ± 0.041	1.796	.074
N (10 g)	93	−0.346 ± 0.038	1.664	.098
N (15 g)	108	−0.576 ± 0.045	5.490	**<.001**
W × N (5 g)	132	−0.322 ± 0.042	1.169	.244
W × N (10 g)	123	−0.281 ± 0.042	0.433	.665
W × N (15 g)	105	−0.570 ± 0.047	4.036	**<.001**
Randomized	144	−0.257 ± 0.038	–	**–**

**Figure 4:**
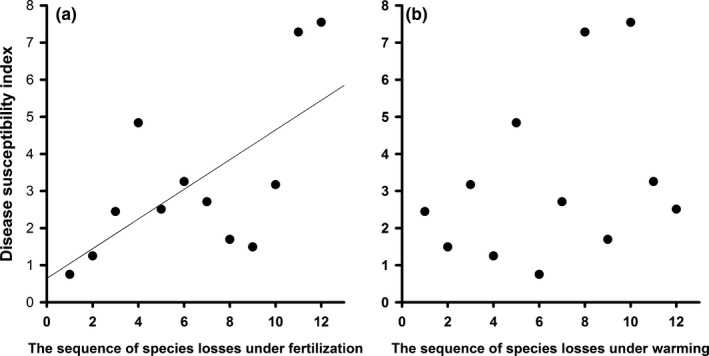
Disease susceptibility index versus the loss sequence for the 12 host species under fertilization and warming, respectively. (a) Disease susceptibility increased linearly with the sequence of species losses under fertilization [information‐theoretic evidence ratio (ER) = 4.23; percent deviance explained (De) = 42.06]; (b) Disease susceptibility was not related to the sequence of species losses under warming (ER = 0.323; De = 11.06)

The disease susceptibility index was positively related to a species’ vulnerability (i.e., its sequence as to when it was lost from the community, as determined by species frequencies among nitrogen treatment plots) under nitrogen addition (ER = 4.23; Figure 4), indicating that disease susceptibility (a species identity effect) drove diversity effects on pathogen load under fertilization. Meanwhile, disease susceptibility was not related to the sequence of species losses under warming (ER = 0.32) or combined warming and fertilization treatments (ER = 0.31; Figure 4).

## 4 DISCUSSION

Our results revealed different effects of species loss orders simulated from fertilization and experimental warming on the host diversity–pathogen relationship compared to random species losses. Based on the simulating approach, random plant species losses might largely underestimated the dilution effect of host diversity on plant foliar fungal diseases in our alpine meadow. Although previous studies have demonstrated that realistic versus random species losses may have different functional consequences in natural and artificial ecosystems (e.g., Bracken et al., 2008; Selmants et al., 2012, 2014; Zavaleta & Hulvey, 2004, 2007), as well as in theoretical models (Ostfeld & Logiudice, 2003), this study investigates how realistic species losses occurring as a result of known drivers (i.e., nitrogen addition and experimental warming) affected the diversity–disease relationship (i.e., dilution effects) compared to random species losses based on the simulating species loss orders.

Community pathogen loads always decreased with increased species richness at our study site. This dilution effect of biodiversity on foliar fungal disease may be attributed to physical isolation of nonhost species. In this specialist pathogen–host system, increasing host species richness was expected to increase the interception of spores by nonhosts (physical isolation), alter microclimatic conditions (e.g., temperature, humidity, illumination, and raindrop splash), and increase spatial heterogeneity as well as three‐dimensional space‐filling capacity, all factors which can ultimately reduce pathogen load in species‐rich assemblages (Liu et al., 2016; Mitchell et al., 2002; Zhu et al., 2000).

We found that realistic species losses simulated from a fertilization treatment produced stronger dilution effects than random species losses; this was similar to the theoretical expectations from studies of Lyme disease, in which dilution effects occur under realistic species losses while amplification effects are expected under random species losses (Ostfeld & Logiudice, 2003). Both their theoretical expectations and our study here showed that the dilution effect was more likely to occur in realistic rather than random species losses. Also, this study demonstrated the influence of losses of particular trait (species disease susceptibility index) on ecosystem functions and services under identified species loss drivers, similar to several previous field‐based studies comparing functional consequences of realistic and random species losses (Bracken et al., 2008; Selmants et al., 2012, 2014; Wolf & Zavaleta, 2015; Zavaleta & Hulvey, 2004, 2007). Further, some researches indicate that species vulnerabilities (to loss) and their contributions to ecosystem functions can be correlated (Taylor, Flecker, & Hall, 2006; Zavaleta et al., 2009), causing disproportionate degeneration of ecosystem functioning when species loss occurs nonrandomly (such as under e.g., eutrophication, drought, and habitat fragmentation).

Disease susceptibility drove the effect of realistic host species loss simulated from fertilization on the biodiversity–disease severity relationship in this study. The disease susceptibility index was positively related to species vulnerabilities to loss under the nitrogen addition treatment. In this case, highly competent hosts likely persisted in low‐diversity assemblages, whereas poorly competent host species only appeared in high‐diversity communities. In contrast to random species losses, community competence under realistic species losses increased disproportionately with decreased species richness under fertilization, which resulted in an enhanced dilution effect of biodiversity on pathogen load (Johnson et al., 2013; Lacroix et al., 2014; Liu et al., 2017). Grassland ecosystems have undergone biodiversity losses worldwide owing to artificial atmospheric nitrogen pollution (Borer et al., 2014). Hence, our results imply that the increased disease risk predicted with vanishing biodiversity in alpine meadows, due to realistic species loss drivers such as nitrogen enrichment, may be more serious than predicted by random species loss experiments.

The positive relationship between a species’ vulnerability under nitrogen addition and its disease susceptibility might result from an evolutionary trade‐off between defense and competitive ability (Huang et al., 2013; Liu et al., 2017; Viola et al., 2010). Specifically, costs to plants of defense against specialist enemies might be higher than for generalist enemies (Joshi & Vrieling, 2005); thus, plant species with a high resilience to disturbance are likely to invest less in defense against pathogens (especially specialist pathogens; Miller, White, & Boots, 2007). Therefore, low competence host species (i.e., those with good defense capabilities) would be the most at risk of loss under nitrogen addition because they are relatively poor competitors under fertilization. For example, species competence against generalist aphid‐vectored viral pathogens was negatively related to their loss order along the North American west coast (Lacroix et al., 2014); this study found a “ubiquity–competence relationship” consistent with that found here, despite our focus on specialist fungal pathogens. Random species loss experiments neglect to account for this relationship and thus underestimate the dilution effect of biodiversity on disease severity in real ecosystems.

In contrast to the experimental nutrient addition treatment, there was no relationship between a species’ disease susceptibility index and the order in which it was lost under artificial warming. Moreover, experimental warming reduced the magnitude of the effect size at each nitrogen addition level, when this fertilization treatment was combined with warming. Analogously, other studies found that heat stress, warming‐induced drought, and litter accumulation, rather than trade‐offs between defense response and competitive ability, were alternative explanations for species loss under artificial warming (as simulated by OTCs) in an alpine meadow (Klein et al., 2004; Liu et al., 2016). Differences in community disassembly rules between fertilization and warming treatments may lead to contrasting effects of realistic species losses on biodiversity–disease relationships.

In conclusion, based on our simulating approach, random species loss experiments may largely underestimate increases in the risk of foliar fungal diseases with biodiversity declines incurred by nitrogen additions. According to our results, careful attention should be paid to modeling diversity–disease relationships in real ecosystems under global change scenarios, and also to distinguishing how various drivers (e.g., nitrogen fertilization and experimental warming) may produce different functional consequences for ecosystems with realistic species losses. Our results also emphasize the need for protection of low competence species in best conservation practices, in order to impede the spread of infectious diseases under global change.

## CONFLICT OF INTEREST

None declared.

## AUTHOR CONTRIBUTIONS

SZ and XL conceived the study. XL, FC, SL and DS collected the data. XL and SZ performed the analyses. XL and SZ wrote the manuscript.
