# Supplementary material for: Random species loss underestimates dilution effects of host diversity on foliar fungal diseases under fertilization
Source: Ecol Evol. 2018 Jan 8;8(3):1705–13. doi: 10.1002/ece3.3749 (PMC5792568; doi:10.1002/ece3.3749)

**Table S1**. Species found in the study site.

| **Family** | **Genus species** |
| --- | --- |
| Alliaceae | *Allium sikkimense* |
| Apiaceae | *Chamaesium paradoxum* |
| Asteraceae | *Ligularia virgaurea* |
| Asteraceae | *Saussurea pulchra* |
| Asteraceae | *Artemisia mongolica* |
| Asteraceae | *Taraxacum mongolicum* |
| Asteraceae | *Anaphalis lactea* |
| Asteraceae | *Saussurea nigrescens* |
| Asteraceae | *Saussurea leontodontoides* |
| Asteraceae | *Ajania tenuifolia* |
| Asteraceae | *Leontopodium haplophylloides* |
| Asteraceae | *Saussurea stella* |
| Asteraceae | *Aster diplostephioides* |
| Caryophyllaceae | *Cerastium fontanum* |
| Caryophyllaceae | *Stellaria uda* |
| Cyperaceae | *Scirpus distigmaticus* |
| Cyperaceae | *Kobresia myosuroides* |
| Cyperaceae | *Kobresia capillifolia* |
| Euphorbiaceae | *Euphorbia helioscopia* |
| Fabaceae | *Astragalus polycladus* |
| Fabaceae | *Oxytropis kansuensis* |
| Fabaceae | *Tibetia himalaica* |
| Fabaceae | *Thermopsis lanceolala* |
| Fabaceae | *Medicago lupulina* |
| Gentianaceae | *Lomatogonium carinthiacum* |
| Gentianaceae | *Gentianopsis paludosa* |
| Gentianaceae | *Halenia elliptica* |
| Gentianaceae | *Gentiana farreri* |
| Geraniaceae | *Geranium pylzowianum* |
| Lamiaceae | *Scutellaria rehderiana* |
| Orchidaceae | *Herminium monorchis* |
| Plantaginaceae | *Plantago depressa* |
| Poaceae | *Poa pratensis* |
| Poaceae | *Elymus nutans* |
| Poaceae | *Agrostis hugoniana* |
| Poaceae | *Koeleria litvinowii* |
| Poaceae | *Stipa aliena* |
| Poaceae | *Festuca sinensis* |
| Polygonaceae | *Rumex acetosa* |
| Polygonaceae | *Polygonum macrophyllum* |
| Ranunculaceae | *Thalictrum alpinum* |
| Ranunculaceae | *Ranunculus tanguticus* |
| Ranunculaceae | *Anemone obtusiloba* |
| Ranunculaceae | *Anemone trullifolia* |
| Ranunculaceae | *Anemone rivularis* |
| Ranunculaceae | *Delphinium kamaonense* |
| Rosaceae | *Potentilla anserina* |
| Rosaceae | *Potentilla bifurca* |
| Rosaceae | *Potentilla potaninii* |
| Saxifragaceae | *Parnassia trinervis* |
| Scrophulariaceae | *Euphrasia regelii* |
| Scrophulariaceae | *Lancea tibetica* |
| Scrophulariaceae | *Veronica eriogyne* |
| Scrophulariaceae | *Pedicularis szetschuanica* |

**Table S2**. Observed diseases and pathogens occurring on the 12 host species used in the removal experiment.

| **Host species** | **Diseases** | **Pathogen species** |
| --- | --- | --- |
| *Anemone rivularis* | black spot | *Alternaria tenuissima* |
|  | leaf spot | Unidentified |
| *Koeleria litvinowii* | leaf blight | Unidentified |
|  | leaf spot | *Ascochyta* sp. |
|  | rusts | *Puccinia recondita* |
| *Elymus nutans* | smuts | *Urosystis dahuricus* |
|  | leaf spot | *Ascochyta* sp. |
|  | rusts | *Puccinia* *recondita* |
| *Festuca sinensis* | leaf spot | *Ascochyta* sp. |
|  | powdery mildew | *Erysiphe graminis* |
| *Potentilla potaninii* | leaf spot | Unidentified |
|  | rusts | *Phragmidium* sp. |
| *Saussurea stella* | brown spot | Unidentified |
|  | rusts | *Puccinia* sp. |
|  | leaf spot | Unidentified |
| *Thermopsis lanceolata* | leaf spot | Unidentified |
|  | rusts | *Uromyces* sp. |
| *Astragalus polycladus* | downy mildew | *Peronospora* sp. |
|  | leaf spot | Unidentified |
| *Anemone trullifolia* | leaf spot | Unidentified |
| *Saussurea nigrescens* | brown spot | Unidentified |
| *Ligularia virgaurea* | brown spot | *Trichometasphaeria* sp. |
| *Kobresia humilis* | leaf blight | Unidentified |
|  | rusts | *Puccinia* sp. |
|  | powdery mildew | *Erysiphe* sp. |

**Table S3**. Examples of species loss orders simulated from fertilization/warming treatments.

| **Species richness = 1** | **Species richness = 2** | **Species richness = 4** | **Species richness = 8** |
| --- | --- | --- | --- |
| **Example 1** |  |  |  |
| *Ligularia virgaurea* | *Ligularia virgaurea* | *Ligularia virgaurea* | *Ligularia virgaurea* |
|  | *Kobresia humilis* | *Kobresia humilis* | *Kobresia humilis* |
|  |  | *Anemone trullifolia* | *Anemone trullifolia* |
|  |  | *Potentilla potaninii* | *Potentilla potaninii* |
|  |  |  | *Saussurea stella* |
|  |  |  | *Saussurea nigrescens* |
|  |  |  | *Koeleria litvinowii* |
|  |  |  | *Festuca sinensis* |
| **Example 2** |  |  |  |
| *Ligularia virgaurea* | *Ligularia virgaurea* | *Ligularia virgaurea* | *Ligularia virgaurea* |
|  | *Kobresia humilis* | *Kobresia humilis* | *Kobresia humilis* |
|  |  | *Anemone trullifolia* | *Anemone trullifolia* |
|  |  | *Potentilla potaninii* | *Potentilla potaninii* |
|  |  |  | *Anemone rivularis* |
|  |  |  | *Saussurea stella* |
|  |  |  | *Thermopsis lanceolala* |
|  |  |  | *Saussurea nigrescens* |
| **Example 3** |  |  |  |
| *Ligularia virgaurea* | *Ligularia virgaurea* | *Ligularia virgaurea* | *Ligularia virgaurea* |
|  | *Kobresia humilis* | *Kobresia humilis* | *Kobresia humilis* |
|  |  | *Anemone trullifolia* | *Anemone trullifolia* |
|  |  | *Potentilla potaninii* | *Potentilla potaninii* |
|  |  |  | *Elymus nutans* |
|  |  |  | *Saussurea stella* |
|  |  |  | *Thermopsis lanceolala* |
|  |  |  | *Festuca sinensis* |
| **Example 4** |  |  |  |
| *Ligularia virgaurea* | *Ligularia virgaurea* | *Ligularia virgaurea* | *Ligularia virgaurea* |
|  | *Thermopsis lanceolala* | *Thermopsis lanceolala* | *Thermopsis lanceolala* |
|  |  | *Anemone trullifolia* | *Anemone trullifolia* |
|  |  | *Potentilla potaninii* | *Potentilla potaninii* |
|  |  |  | *Saussurea nigrescens* |
|  |  |  | *Koeleria litvinowii* |
|  |  |  | *Festuca sinensis* |
|  |  |  | *Astragalus polycladus* |
| **Example 5** |  |  |  |
| *Ligularia virgaurea* | *Ligularia virgaurea* | *Ligularia virgaurea* | *Ligularia virgaurea* |
|  | *Thermopsis lanceolala* | *Thermopsis lanceolala* | *Thermopsis lanceolala* |
|  |  | *Anemone trullifolia* | *Anemone trullifolia* |
|  |  | *Potentilla potaninii* | *Potentilla potaninii* |
|  |  |  | *Koeleria litvinowii* |
|  |  |  | *Festuca sinensis* |
|  |  |  | *Astragalus polycladus* |
|  |  |  | *Anemone rivularis* |

**Figure S1.** Photograph of the removal experiment. Photo credit: Xiang Liu.


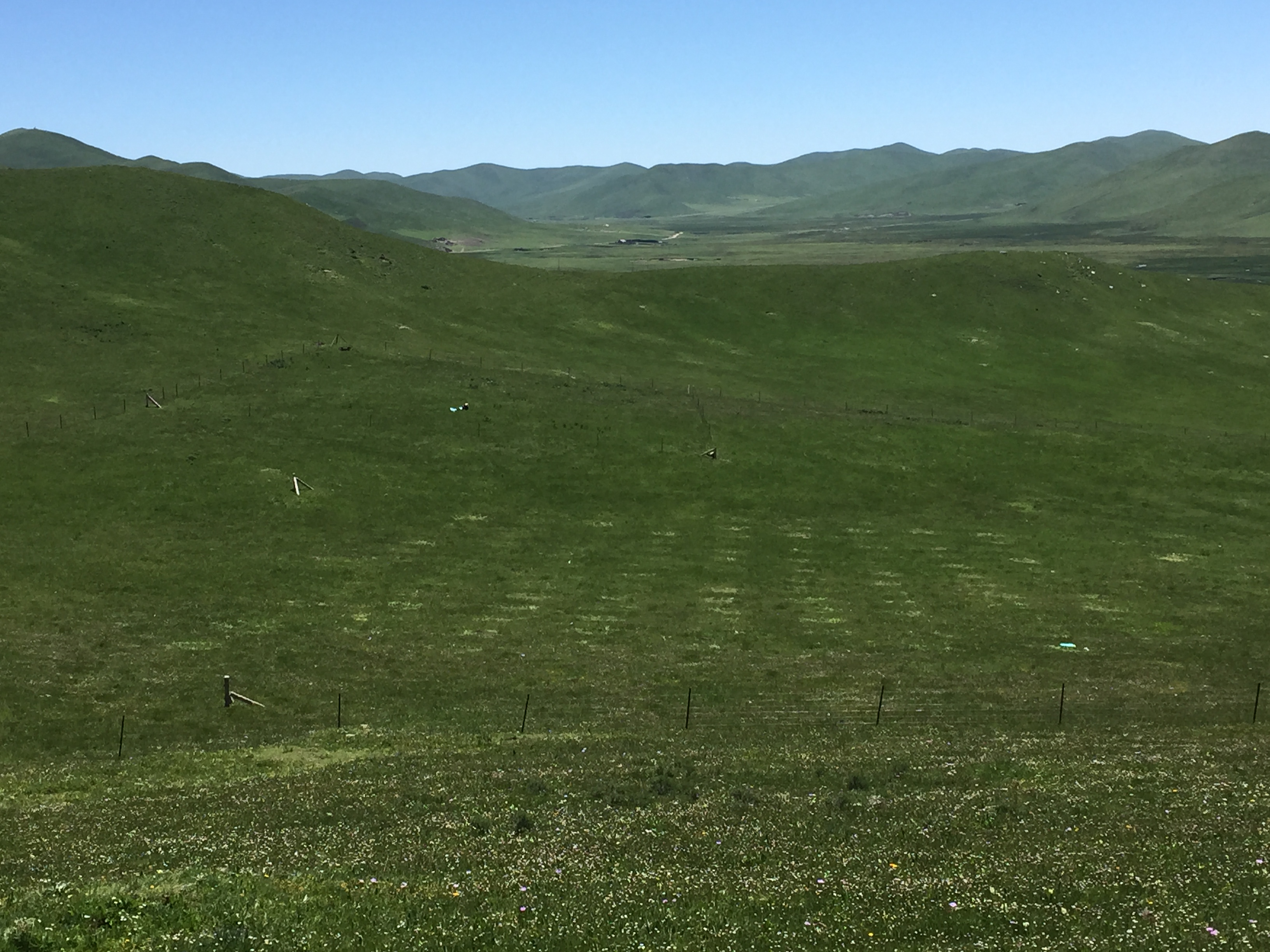

Supplement: Supplementary file 1 [file ECE3-8-1705-s001.doc]
